# Diagnosis of Depressive Disorder Model on Facial Expression Based on Fast R-CNN

**DOI:** 10.3390/diagnostics12020317

**Published:** 2022-01-27

**Authors:** Young-Shin Lee, Won-Hyung Park

**Affiliations:** 1 Department of Nursing, Fareast University, Eumseong 27601, Korea; florence@kdu.ac.kr; 2 Department of Information Security Protection Engineering, Sangmyung University, Cheonan 31066, Korea

**Keywords:** fast R-CNN, depressive disorder, deep learning, diagnosis, facial expression

## Abstract

This study examines related literature to propose a model based on artificial intelligence (AI), that can assist in the diagnosis of depressive disorder. Depressive disorder can be diagnosed through a self-report questionnaire, but it is necessary to check the mood and confirm the consistency of subjective and objective descriptions. Smartphone-based assistance in diagnosing depressive disorders can quickly lead to their identification and provide data for intervention provision. Through fast region-based convolutional neural networks (R-CNN), a deep learning method that recognizes vector-based information, a model to assist in the diagnosis of depressive disorder can be devised by checking the position change of the eyes and lips, and guessing emotions based on accumulated photos of the participants who will repeatedly participate in the diagnosis of depressive disorder.

## 1. Introduction

With the emergence of a digital approach to mental health, artificial intelligence (AI) and machine learning have been developed and used as prediction, monitoring, and treatment methods to manage mental health. During the coronavirus disease 2019 (COVID-19) pandemic, this digital approach is particularly significant as it enables the protection of medical service users and providers. The digital mental health field is already firmly established with continuous and new research on AI-led solutions for mental health [1]. Various AI technology interventions in mental health allow a full approach beyond diagnosis to provide personalized interventions and feedback based on the results. Moreover, it is possible to provide mental health assessment and intervention through virtual reality [2], and the response of brain waves to visual stimulation can be evaluated through stimulation transmission and leave wave measurement to supplement the diagnosis of dementia and depressive disorders [3]. However, because special equipment is required, they are generally difficult to apply.

Compared to special equipment that is difficult to distribute to the general public, smartphones have the advantage of being distributed to relatively many people, as well as managing ecological momentary assessments (EMA) for mental health monitoring to easily and efficiently send repeated questionnaires [4,5,6]. Ecological momentary intervention (EMI), a new idea that goes beyond simple evaluation and maintains ecological momentality, is also possible. EMI is identifiable by responses to EMA, such as providing instantaneous psychological interventions or behavioral prompts delivered through personal mobile devices during an individual’s daily life [7,8,9].

The World Health Organization sees depressive disorder as an extremely common psychiatric problem with 5.0% of adults worldwide suffering from it and they regard it as a major cause of the global burden of disease (WHO, 2021) [10]. Depressive disorder cannot be overlooked because it can lead to suicide. Even if it starts with mild depression, untreated depression can lead to severe dysfunction or suicide. Treatments can be applied by dividing them into mild, moderate, and severe, and primary prevention and early intervention are effective. Responses of self-report questionnaires [11,12,13], such as self-reporting test tool Patient Health Questionnaire 9 (PHQ-9), can be used as data for the identification of people with depressive disorders. However, it is difficult to confirm only using self-reporting test tools; it is necessary to collect various comprehensive data on areas such as the participant’s mental state and spiritual function. In addition, the characteristics of depressive symptoms by life cycle should be identified to take into account the characteristics of symptoms by age [14]. The Diagnostic and Statistical Manual of Mental Disorders (DSM-5) provides reference criteria for diagnosing depressive disorders by psychologists, and the PHQ-9 scale is also based on DSM-5 criteria for diagnosing depressive disorders. Digital technology-based diagnosis of depressive disorders has a way to respond to self-report surveys through smartphone applications, and analysis to detect depression based on touch typing is also possible.

Initial studies have been conducted to identify schizophrenia or mental illness through digital devices [15,16]. Through sleep state tracking using smartphones and wearable devices, depressive symptoms, anxiety, and psychological problems can be identified, and appropriate interventions can be linked based on them [17]. Studies are also being conducted to apply virtual assistants and digital technology to evaluate the cognitive decline of the elderly [18], patient intake and referral support [19]. In addition, recommendations for personalized treatments can be delivered through AI [20]. Various wearable devices are being developed and distributed but there is a large difference in price and function, making it difficult to purchase and apply them; the spread of wearable devices has not reached the level of smartphone distribution. To confirm the mood in mental state assessment, facial expressions can be examined [14]. Facial expressions convey certain emotions, providing critical information; the degree of agreement between subjective skills and facial expressions should also be checked. Facial expressions can be collected using a smartphone’s camera, and that information can be used for the diagnosis of depressive disorders, along with the results of a self-report questionnaire. In this way, there are various approaches to check mental state, but studies that help diagnose depressive disorders using AI tools are insufficient.

Therefore, this study proposes the development of a depressive disorder diagnosis assistance system using a real-time object recognition chatbot by detecting individual smartphone users. The proposed system can recognize facial expressions using a smartphone camera and uses KakaoTalk’s chatbot platform to increase accessibility. Fast R-CNN can be used for deep learning to recognize the facial expressions associated with emotions caused by depressive disorders. Kakao i Open Builder Platform can be used to provide chatbot services, and cloud server construction will be required for deep learning. The system can update new information through chatbots and enable constant management. After receiving a response to a self-report questionnaire, counseling and resource connections can be used as chatbot services to provide intervention services at the time of diagnosis.

### 1.1. Detecting Individual Smartphone Users and Supporting Mental Health through Chatbots

User detection [21] or digital phenotyping [22] refers to inferring context and behavior information about an individual using sensors and data usage on a smartphone, and predicting psychological results and mental health using machine learning. In addition, previous studies conducted on detecting schizophrenia symptoms using wearable devices, such as smartwatch devices, found that the number of text messages as well as the number and duration of outgoing calls are associated with the recurrence of schizophrenia [23,24]. There was also an initial discussion on the use of Internet of Things for mental health [25]. User movement and physical activity tracked by geographic location and accelerometer sensors, and keystroke dynamics such as clicking, tapping, scrolling, and swiping, especially in terms of smartphone screen input, providing clues to mental health, can help collect information on depressive and anxiety symptoms [26]. Such detection has a high possibility of providing accurate information about mental health, but the user is unaware of the detection to confirm the disorders. Therefore, various attempts and verification are required until implementation.

However, recognizing facial expressions through camera photographed images and checking emotions can be easily collected and used. In the 1960s and the 1970s, American psychologist Paul Eckman saw that human emotional expression is common worldwide, so emotional state reasoning through facial expressions is reliable [27,28]. Currently, many researchers question the evaluation of facial expressions. This is because facial expressions are much more complex than expected, and expressions may vary from culture to culture [29]. The AI Now Institute Research Center at New York University called for a ban on the use of emotional recognition technology in sensitive situations such as hiring or law enforcement [30]. Emotions cannot be completely verified through expression, thus, changes in skin tone can be connected to emotions [31]. It was observed that a visual context, such as a background scene, became a clue to the emotional state [32]. A method has been developed in which users enter their various facial expressions and AI extracts them into six emotions based on the input content [33]; if the reasoning of the emotional state is based on individual users’ data, more accurate confirmation of emotions is possible.

Chatbots are computer programs that implement conversations, where users observers talk to the user through a text or voice-based chat interface. ELIZA, a text-based interactive system developed in MIT in 1966, diagnosed disorders through sentences entered by users with Rogers’ psychotherapy role. Moreover, voice recognition technology has existed since the 1950s, and now chatbots under various conditions have been developed for diagnosing various disorders, such as depressive disorders, autism, and those related to anxiety. User satisfaction with chatbots is high, and their efficacy is being confirmed [34,35]. Chatbots help search for data simply guide researchers on how to use the recommended system interface. After a simple interactive interaction, it is connected to related mental health information or treatment content.

It is not easy to create AI at a level similar to that of a real human therapist, but if an AI agent that integrates sophisticated natural language processing can implement conversational techniques using therapeutic techniques, it may come close. However, the biggest advantage is that chatbots are provided in their own medium through interaction with individual users rather than for the purpose of developing to replace human therapists. It can be used by people who have experienced discomfort in psychiatric diagnosis and treatment or are concerned about stigma. Chatbots that have recently appeared in relation to mental health include Warbot [36], Shim [37], Wisa [38], and Tes [39]. Warbot provides cognitive behavioral therapy in the form of simple daily conversations and mood tracking to help customers with symptoms related to depression and anxiety.

### 1.2. AI-Based Depressive Disorder Diagnosis

Depressive disorder makes it difficult for the affected individuals to perform meaningful daily activities—such as sleeping, eating, thinking, and bodily functions—beyond feeling depressed [14]. The evaluation of depressive disorder is divided into “clinic-rated” and “self-report” [40]. In psychology, self-reported evaluation scales have generally been widely accepted and are viewed as cost-effective means [41]. Individual subjective appeals are the most crucial information on the assumption that the participants are responding honestly. Items that can detect users participating in self-report evaluations responding falsely may be used to increase the reliability of the test [42]. Owing to the reliability of this self-reported evaluation scale, it is observed that the introduction and utilization of digital methods makes it easy to diagnose mental health problems. The most commonly used self-reported evaluation scale tools include the Beck Depression Inventory (BDI), Patient Health Questionnaire (PHQ)-9, and the Zung Self-rating Depression Scale (ZSDS) [14].

**Figure 1:**
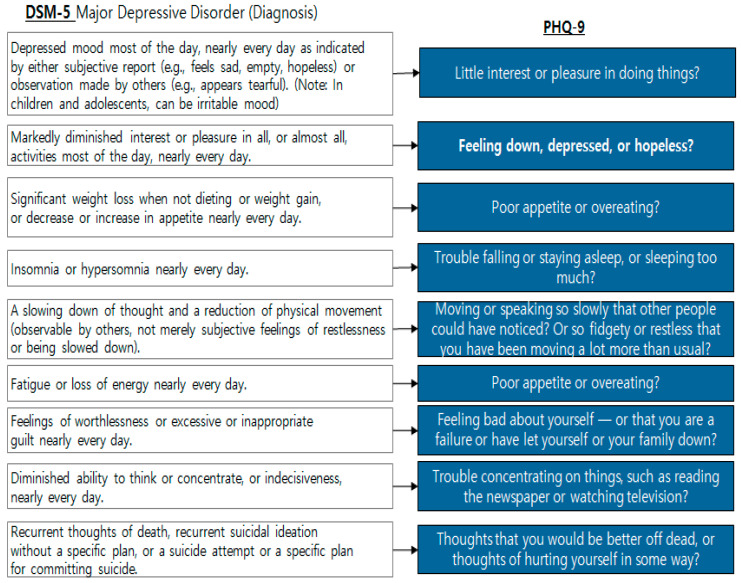
DSM-5 [44] and PHQ-9 [43].

PHQ-9 [43] consists of nine questions for the diagnosis of depressive disorder according to the diagnostic criteria for the Diagnostic and Statistical Manual of Mental Disorders (DSM-5) [44], which can simply screen depressive symptoms and evaluate their severity (Figure 1). The PHQ-9’s total score ranges from 0–27, with mild depression over 5, moderate depression over 15, severe depression over 19, and a higher score indicating greater severity [43].

**Figure 2:**
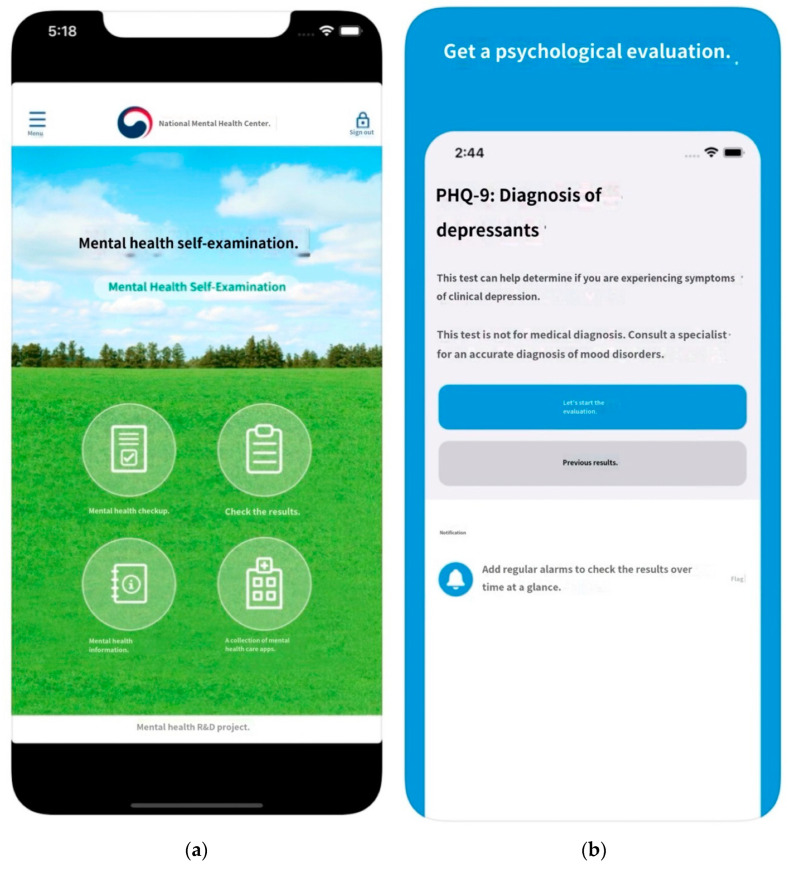
Examples of applications that provide self-diagnosis services for depression through PHQ-9: (**a**) National Mental Health Center [47], (**b**) Inquiry Health LLC [48].

PHQ-9 has proven to be valid and useful in many studies, so it has been translated into various languages and widely used [45]. An application [46,47] for self-report diagnosis and intervention was developed based on the Korean version of the depression screening tool (PHQ-9) [48] (Figure 2).

However, clinical evaluation and self-report questionnaires are crucial in evaluating mental health problems which can be conducted through interviews with patients and their neighbors by psychiatrists. Interviews can be confirmed through mental state examination, such as overall appearance including hygiene, eye contact with others, speed of speech, voice volume, and short or lack of answers to questions [14]. In addition, evaluation of mood, thinking, perception, cognition, and suicide assessment can be performed, and it is necessary to confirm facial expressions that cannot be verified through a self-report questionnaire.

**Figure 3:**
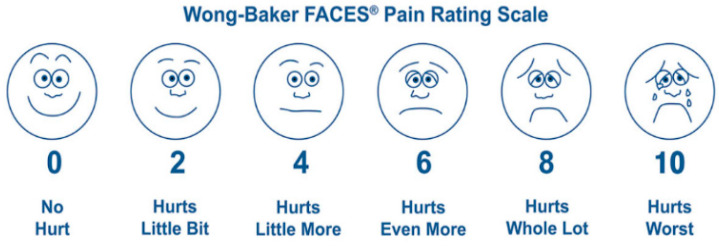
Wong–Baker’s facial pain measurement tool [49].

**Figure 4:**
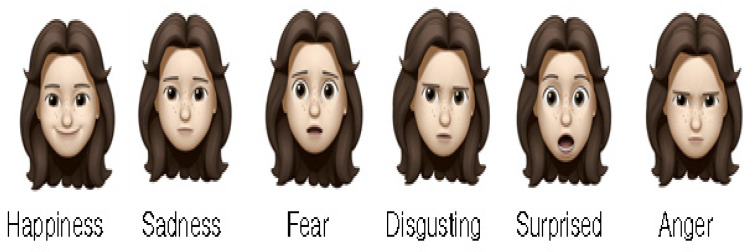
Example of a facial expression that shows the user to select an expression close to his or her emotions [50].

In collecting information on facial expressions, using a self-reported method allows the selection of an expression that is considered closest to the current expression. Wong-Baker’s facial pain measurement tool, presented as a facial expression picture to determine the degree of pain in patients complaining of it, is a reliable tool that believes can inform people aged ≥ 3 years of the level of pain expression [49] (Figure 3). Similar to a facial pain measurement tool, an expression can be presented to a user and verified that they believe it to be you’re their current expression [50] (Figure 4).

However, subjective self-report has limitations and checking facial expressions by evaluators at clinical sites is crucial information in reviewing mood and degree of agreement subjectively described by clients with depressive disorders. Therefore, objective information on facial expressions is needed. Checking facial expressions through software has led psychologists to disagree whether facial expression recognition through software is reliable, as they vary from person to person and various emotions can appear in combination [29]. Comparing facial expressions of the same user may provide meaningful information because it involves the same condition other than the variable of mood state change. The technology to determine emotions using the CNN algorithm by adding various facial expressions to be recognized and extracting features for the entire image from the input facial image can assist in the diagnosis of depressive disorder.

## 2. Materials and Methods

**Figure 5:**
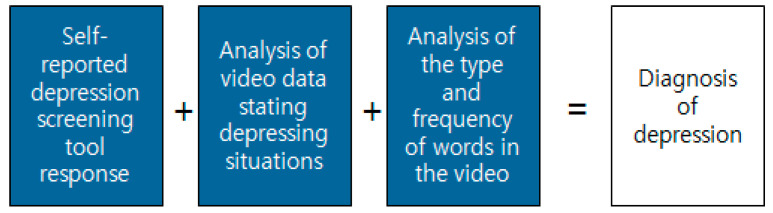
Conceptual diagram of service of the proposed system.

Figure 5 shows the service conceptual diagram of the emotion recognition system for assisting in diagnosing depressive disorders. Initially, the user accesses the chatbot to diagnose depressive disorder. After responding to the PHQ-9 questionnaire supported by voice, the response results are transmitted as scores, and the connectable resources are guided according to the score level. For emotional diagnosis, the user takes a picture of their face and sends it to the chatbot. The picture is transmitted from the chatbot server to the flask web server built on the cloud. It distinguishes emotions by recognizing objects using deep learning models within the server. Then, the result is transmitted to the chatbot, which delivers the emotion determination result to the user. Because this system must create a deep learning model that can recognize objects in the video in real time, it selects a deep learning algorithm as well as collects image data and learns data for model learning. Furthermore, it builds a deep learning server to use the model and performs a KakaoTalk linkage process with the server.

### 2.1. Deep Learning Algorithm Selection

The deep learning algorithm uses fast R-CNN [51], through which a feature map is extracted by sequentially applying convolution, linear rectification, and max pooling processes from facial images, and a region proposition network (RPN) is learned from them. Eye and lip detectors are learned using the proposed area and a feature map. Male and female Korean face images were used for learning. In a study that detected eye and lip regions from facial images using Fast R-CNN, the average accuracy was 97.7% for eyes and 91% for lips [52].

It is performed by finding the parameters *W* and *b* of Fast R-CNN, and it can be expressed as Equation (1). The loss compensation module operates by being weighted with a hyper-parameter λ, and as shown in Equation (2), by optimizing the parameters of the domain classifier, that is, weight (*u*) and bias (*z*), it is possible to minimize the domain discrimination loss and maximize the distance between domains. In other words, the parameters of Fast R-CNN are optimized in the direction of extracting features that can distinguish the two domains.

(1) $$\underset{W,b}{min}\left[\frac{1}{n}\overset{n}{\underset{i=1}{\sum }}L_{det}^{i}\left(W,b\right)+\lambda R\left(W,b\right)\right]$$

(2) $$R\left(W,b\right)=\underset{u,z}{min}\left[\frac{1}{n}\overset{n}{\underset{i=1}{\sum }}L_{dom}^{i}\left(W,b,u,z\right)+\frac{1}{n^{\prime }}\overset{n}{\underset{i=n+1}{\sum }}L_{dom}^{i}\left(W,b,u,z\right)\right]$$

### 2.2. Collecting and Learning Image Data

Image data will use internet collection and direct collection. First, shareable data among the data used in the development of facial expression recognition is used. Since this system proposes a method of comparing and checking based on the user’s accumulated facial expressions, direct collection is performed by photographing various facial expressions from the same person through direct photographing. Algorithmic suggestions to detect the position of the eyes and lips have been represented in the block diagram by Lee Jung-hwan [52].

To create a deep learning model, data learning of the collected images must be performed. A 14×19-sized map was used to detect eye and lip regions from data collected in the study by Lee Jung-hwan [52], and the number of channels was 32. The background area and the entire management area were classified by passing through the convolutional neural network, and the eye or lip area was searched using an anchor box and a ground-truth box. Fast R-CNN learning has four steps. The first step is to learn the RPN using learning data; second, the detector is learned using the suggested area obtained from the RPN; third, the weights of the convolution and max pooling steps of the block diagram are shared with each other [52].

### 2.3. Connect KakaoTalk with the Server

When a user sends a picture to a chatbot, there is a need for a server to perform deep learning that can determine the picture. In this study, cloud integrated development environment (IDE) was selected. Cloud servers are price-competitive and have easy server expansion, making it easy to respond to traffic congestion and strong security. The Flask web server will be installed on the cloud server so that chatbots can provide appropriate answers to facial expressions; an emotional recognition judgment model that recognizes emotions from facial expressions as much as tip learning is used in the cloud server. Afterwards, chatbots will be developed to provide services for diagnosing depressive disorders using Kakao i Open Builder provided by Kakao. The Kakao i Open Builder consists of a plug-in that provides the answers desired by chatbot workers for certain functions, a user’s expected question that can draw out patterns created by bot workers, and skills created by bot workers not provided by Kakao i.

## 3. Depressive Disorder Model on Facial Expression

**Figure 6:**
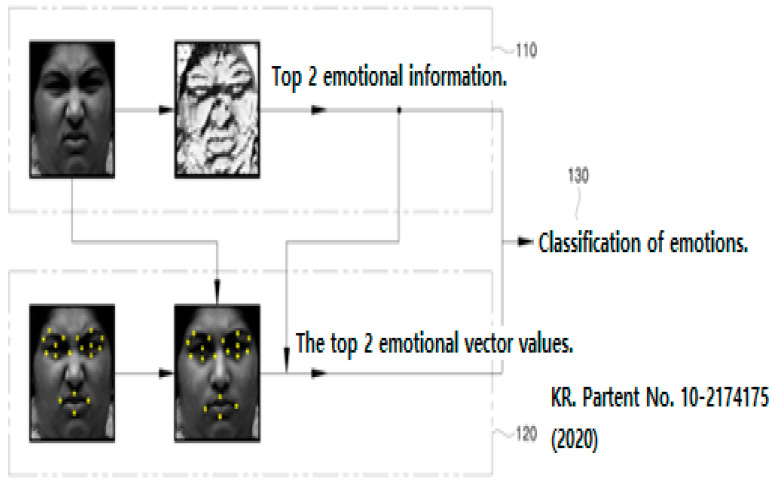
Facial emotion recognition device and method for identifying emotions [33].

**Figure 7:**
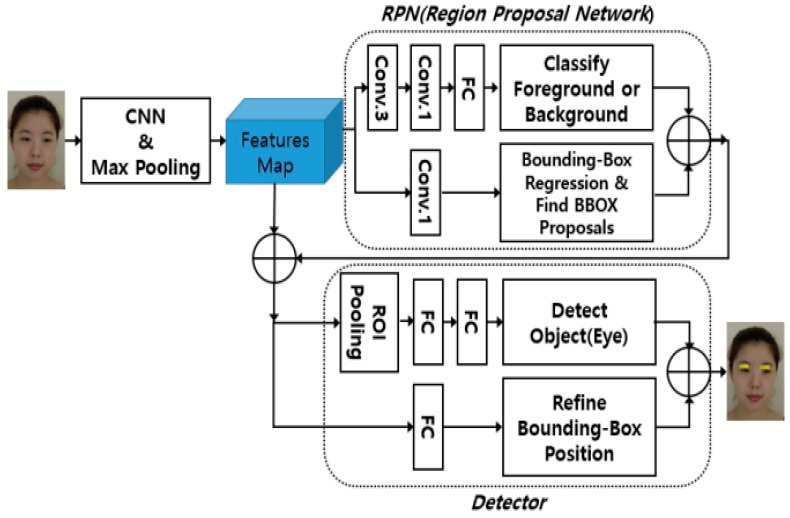
Block diagram for detecting the position of eyes and lips proposed in the study of Lee Jeong-hwan (2018) [51].

**Figure 8:**
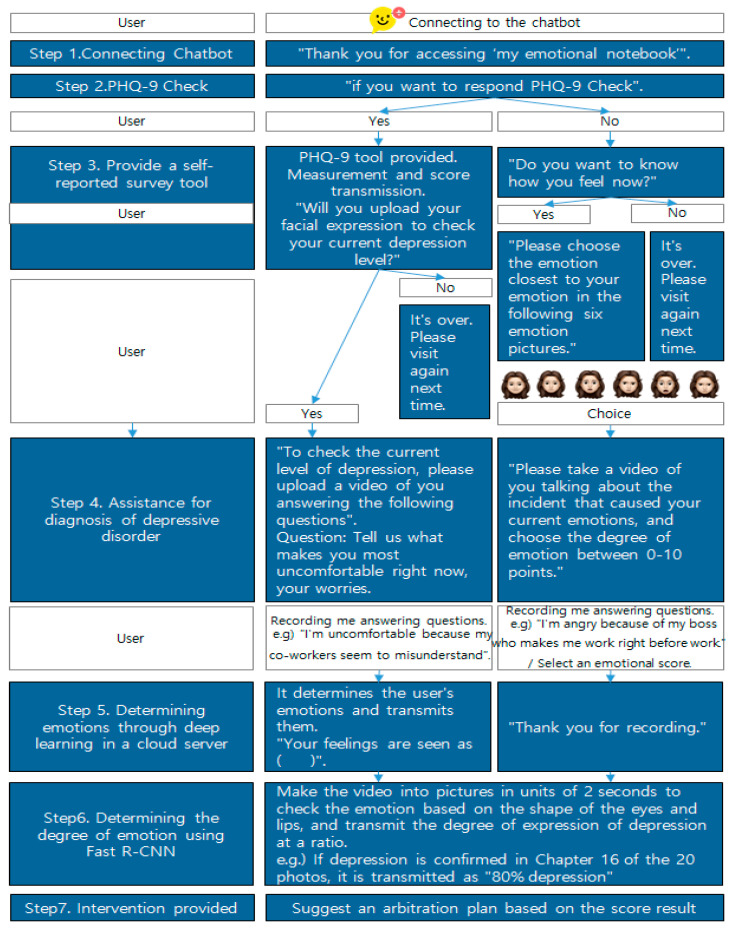
Suggestions to assist in diagnosing depression using a chatbot.

Existing technical parts can be used to determine emotions (Figure 6 and Figure 7). Through deep learning of Kakao’s chatbot and cloud servers, you can receive videos containing emotional expressions from users, analyze photos extracted from multiple pieces of video data, and express the degree to which emotions are revealed in proportion. The most important point is that it is possible to more accurately grasp the user’s emotions by accumulating image data input by the user (Figure 8). 

The proposed depressive disorder diagnosis assistance system that uses the real-time object recognition chatbot enables real-time emotional evaluation using smartphone camera images and is expected to increase user convenience through KakaoTalk’s chatbot platform. However, there is opposition from a group of cognitive scientists to whether facial expressions can detect emotions, and the risk of using facial recognition information through AI for criminal search is possible, but human expressions reveal emotions that are beyond culture. Nevertheless, there are differences in the way emotions are expressed across cultures, and human emotions are complex and subtle; thus, various emotions are felt concurrently. Emotions can be understood only by viewing the background and presentation of the rest of the body. Therefore, it is expected that the comparative analysis method of emotion recognition through facial expressions—based on the data collected for each user—can increase the accuracy of emotion recognition.

The proposed depressive disorder diagnosis assistance system that uses the real-time object recognition chatbot enables real-time emotional evaluation using smartphone camera images and is expected to increase user convenience through KakaoTalk’s chatbot platform. However, there is opposition from a group of cognitive scientists as to whether facial expressions can detect emotions, and the risk of using facial recognition information through AI for criminal search is possible, but human expressions reveal emotions that are beyond culture. Nevertheless, there are differences in the way emotions are expressed across cultures, and human emotions are complex and subtle; thus, various emotions are felt concurrently. Emotions can be understood only by viewing the background and presentation of the rest of the body. Therefore, it is expected that the comparative analysis method of emotion recognition through facial expressions—based on the data collected for each user—can increase the accuracy of emotion recognition.

## 4. Conclusions

In this study, the model was limited to the field of the depressive disorder diagnosis assistance system, but smartphones have a greater potential for EMI. When the level of detection through smartphones is further improved and insight into digitally identified expressions increases, it can be integrated to communicate personalized treatment recommendations through AI. Voice chatbots have already been implemented in innovative remote health solutions using medical services during the COVID-19 pandemic. Automatic acute treatment classification and chronic disease management are possible, including remote monitoring, preventive management, patient intake and referral support. Although it was not included in this study, it is possible to receive help in diagnosing depression through Natural Language Processing with an AI. AI-based chatbots can respond to new inputs that do not exist through deep learning, and conversations that fit the context are possible considering the contents of previous conversations. However, it requires a minimal additional other than the cost of development, such as for digital treatments. In addition, the deployment time and cost can be reduced when expanding to other domains. The proposal for arbitration is also thought to improve the benefits of various dimensions related to mental health by considering methods with high accessibility, such as Kakao’s chatbot.

## Data Availability

Not applicable.
